# Evaluation and facilitation of intervention fidelity in community exercise programs through an adaptation of the TIDier framework

**DOI:** 10.1186/s12913-020-4919-y

**Published:** 2020-01-30

**Authors:** Marie-Louise Bird, William B. Mortenson, Janice J. Eng

**Affiliations:** 1 Department of Physical Therapy, Faculty of Medicine, University of British Columbia, 212-2177 Wesbrook Mall, Vancouver, BC V6T 1Z3 Canada; 2 Rehabilitation Research Program, GF Strong Rehabilitation Research Lab, Vancouver Coastal Health Research Institute, 4255 Laurel Street, Vancouver, BC V5Z 2G9 Canada; 3 Department of Occupational Science and Occupational Therapy, Faculty of Medicine, University of British Columbia, T325 - 2211 Wesbrook Mall, Vancouver, BC V6T 2B5 Canada

**Keywords:** Implementation, Fidelity, Framework, Adaptation, Evaluation, Program, Scale-up

## Abstract

**Background:**

Despite high quality evidence supporting multiple physical and cognitive benefits of community-based exercise for people after stroke, there is little understanding on how to facilitate uptake of these research findings to real-world programs. A common barrier is a lack of standardised training for community fitness instructors, which hampers the ability to train more instructors to deliver the program as it was designed. Scaling up program delivery, while maintaining program fidelity, is complex. The objective of this research is to explore novel use of the Template for Intervention Description and Replication (TIDier) framework to evaluate and support implementation fidelity of a community exercise program.

**Methods:**

We embedded intervention fidelity evaluation into an inaugural training program for fitness instructors who were to deliver the Fitness and Mobility Exercise Program for stroke, which has established efficacy. The training program consisted of a face-to-face workshop followed by 3 worksite ‘audit and feedback coaching cycles’ provided over 3 iterations of the 12-week program offered over 1 year. A modified TIDIER checklist (with 2 additional criteria) was used within the training workshop to clarify the key ‘active ingredients’ that were required for program fidelity, and secondly as a basis for the audit and feedback process enabling the quantitative measurement of fidelity. Data were collected from audits of observed classes and from a survey provided by fitness instructors who implemented the program.

**Results:**

We demonstrated the feasibility of the TIDier checklist to capture 14 essential items for implementation evaluation of a complex exercise intervention for people with chronic health conditions over 3 iterations of the program. Based on the audit tool, program fidelity was high and improved over time. Three content areas for workplace coaching (intensity monitoring, space, and educational tips) were identified from the audit tool and were addressed.

**Conclusion:**

Training of staff to deliver exercises to high need populations utilising workshops and workplace coaching that used the TIDier framework for training, onsite audit and feedback resulted in a high level of fidelity to the program principles. A novel checklist based on the TIDier framework was useful for embedding implementation fidelity in complex community-based interventions.

## Background

While there is good quality evidence and guidelines supporting community-based exercise to improve physical and cognitive functioning in people with stroke [1], there is little understanding on how to move these programs from research to real-world programs [2]. Implementing community-based exercise programs for people with chronic health conditions like stroke is a complex process. Elements of complexity include multiple active ingredients in the exercise program (e.g., strengthening, aerobic, balance exercises); specialised training to develop multiple skills for community staff who do not typically work with people with chronic disease who may be more frail with serious physical and cognitive challenges; and organisational change to refer stroke patients to community settings for exercise [3].

The lack of ability to standardise the delivery and evaluation of complex health interventions can arise in barriers to implementation [4] and jeopardize the effectiveness of the interventions when delivered in a ‘real life’ setting. There is a natural tension between the goal of implementation fidelity (i.e., ‘the degree to which programs are implemented as intended by the program developers’ [5]) and the need for local contextualisation (i.e., adapting the program to the local setting [6]) [7]. Program developers and implementers must be very clear about the ‘active ingredients’ that are the essential elements that a newly established program must include for that program to be considered implemented with fidelity to the original [8].

Implementing community-based exercise programs remains challenging [9], with a lack of published information on how to provide local contextualisation while keeping fidelity to essential elements [10]. Developing methods to assist community-based organizations to uptake established exercise evidence to routine practice while maintaining fidelity may be the most helpful way to advance decision making in this area.

The objective of this research is to evaluate implementation fidelity of a complex multi-component community-based exercise program using a framework adapted from the Template for Intervention Description and Replication (TIDier) checklist that we embedded in a training program built on the TIDier framework when we ran it for the first time. The intervention implemented was the Fitness and Mobility Exercise (FAME) for people after stroke which has a high level of effectiveness that had been demonstrated in multiple randomised controlled clinical trials [11–13].

## Methods

### Setting and study design

**Fig. 1:**
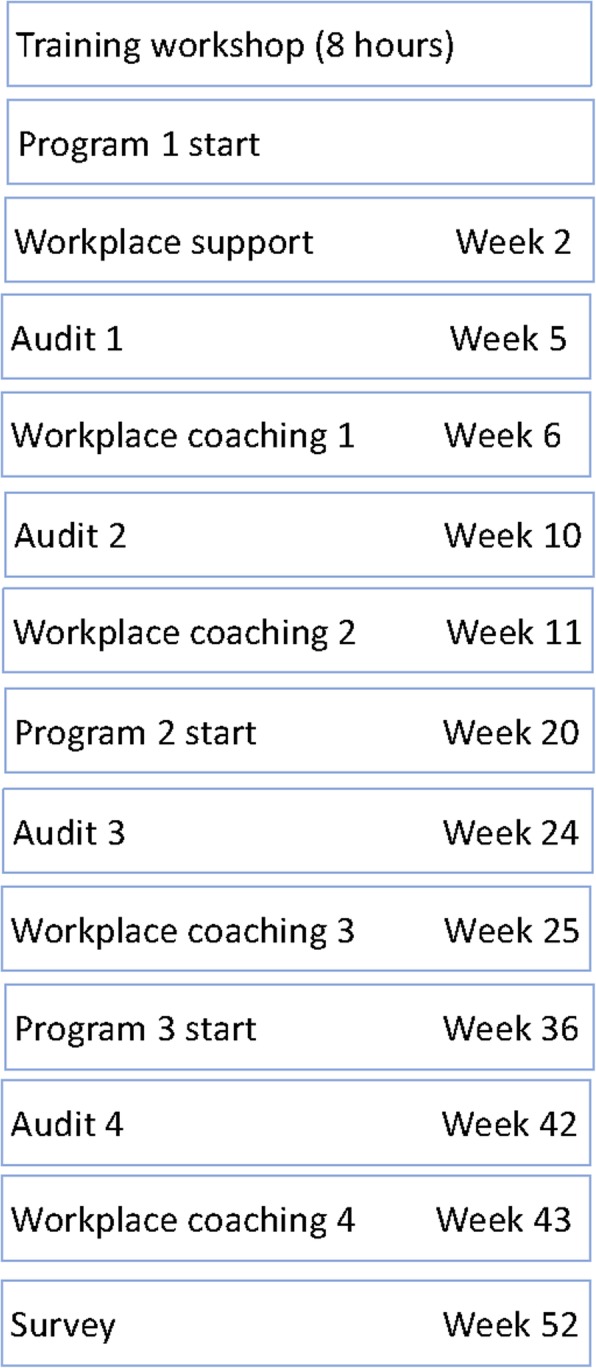
Study design timeline

This project took place in a recreation community centre in a suburb of a large metropolitan city. The staff to deliver the program were fitness instructors employed at this centre who had experience in delivery of group classes to older adults but had no experience with stroke. Training consisted of a one-day workshop, four workplace audits and four sessions of workplace coaching 1 week after each audit over three iterations of the program over 1 year (Fig. 1). All the training was delivered physical therapists. One physical therapist visited during week two to support the fitness instructors and answer any questions they had. This project was granted institutional approval by The University of British Columbia Office of Research Services Behavioural Research Ethics Board (H16–03292-A003).

### Intervention FAME exercise program

#### Exercise program

The exercise program that was delivered was the FAME program [11, 12]. The FAME exercise program is a community-based circuit style exercise program for with a warm up, exercise stations to improve balance, functional strength and fitness, followed by a cool down stretch session. The program ran for 12–16 weeks each time, in line with the community centre schedule and was delivered three times over the year. The classes were delivered twice a week with up to eight participants with stroke and two instructors. The high level of supervision is a unique aspect to accommodate frailer, fall-prone participants with serious physical and cognitive challenges in a community setting.

### Intervention workshop and workplace coaching

Two physical therapists with FAME experience facilitated a workshop which consisted of 3 h of lectures, 3 h of practical with 3 people with stroke and 2 h of discussion and evaluation. Fitness instructors who signed up for the workshop were then given the option to provide informed consent to using their survey and feedback data for research purposes (all agreed). All teaching resources and manuals are available from FAMEEXERCISE.com. To improve the fidelity of the program delivery, several tools were introduced to the fitness instructors for later use including reminders of the core components (listed on a bookmark), intensity monitoring charts and images of all exercises. These tools included an outline of the essential elements (described here as program principles) and fitness instructors were informed that they would receive feedback after auditing based on 7 principles: Progression, Repetition, Intensity, Normal movement, Core components, Encouragement and education (PRINCE) (see Table 1 for description).

**Table 1:** Program Principles (Acronym PRINCE)

Principle	Description
Progression	The body needs to be continually exposed to challenges. Increase repetitions then difficulty. Stop if overexertion is seen.
Repetition	High repetitions are need for brain remodelling, improving strength and endurance
Intensity monitoring	Exercise should be performed at low to moderate intensity. Intensity should be monitored.
Normal movement patterns	Normal movement should be encouraged, but compensations should not prevent participation unless painful or unsafe.
Core components (FAB)	Functional strengthening Agility and fitness Balance
Encouragement and education	Choose activities for participant success and offer verbal encouragement. Remember to discuss educational tips to encourage self-management.

### Workplace coaching for fitness instructors

All fitness instructors who regularly delivered the FAME program over the first year participated in the workplace audit and coaching process. The workplace coaching was facilitated by one of the physical therapy instructors who had delivered the day-long workshop. The content of each coaching session was determined by the workplace audit which took place a week before each of the coaching sessions.

### Audit

The audit tool was based on the format of the TIDier framework checklist [14] and included the 12 original TIDier item headings to evaluate the extent to which the program principles were included in the observed delivery of the program (Additional file 2: Table S1) and described the program expectations in enough detail to measure fidelity. Two new items were added reflecting the use of the checklist as an audit tool; the first relating to pre-delivery items and the second to identify areas to give feedback. Two physical therapists who were experienced with delivering the FAME Program to people with stroke developed the checklist and came to consensus on the adaptation from the more general TIDier checklist to the more specific stroke-exercise specific items. The checklist was tested in a sample class. Two physical therapists (the same as those who facilitated the original workshop) completed the first audit and one therapist completed the tool independently at the subsequent assessments. Verbal feedback was given to the instructors immediately after the class. In addition, a written summary was emailed to the program manager, who discussed the results with staff. The auditors took a formative assessment approach and used feedback from the audits to inform the subsequent workplace coaching sessions. This resulted in one-hour coaching sessions that were held the next week after the audit and immediately after the class.

### Survey and feedback

After 12 months and three rounds of the FAME program, the fitness instructors were invited to complete a closed online survey to indicate how they had used the training in their practice and what other training needs there were. The survey used open-ended questions to ask about the fidelity to the program content and delivery, challenges and future training needs. The survey also included a series of open-ended Likert scale questions to determine the impact of the workplace coaching. The survey was administered online using a Qualtrics platform through an anonymous email link. The survey was open between 24.04.2018 and 24.06.2018. Instructors were able to go back and change responses. The survey collected IP address data so that multiple entries could be monitored. All instructors received a $CAD25 gift card after the survey was completed. The CHERRIES checklist was used in designing the online survey [15]. A copy of the survey is included as Additional file 1.

### Data analysis

An inductive form of content analysis was used explicitly to categorise open-ended survey responses on training and implementation fidelity [16]. The data was reviewed iteratively to find commonality and patterns across responses to the open-ended questions. The initial analysis was performed by a researcher with a clinical (physical therapy) background and qualitative research experience in implementation, and then reviewed by all team members. Quotes from individual instructors were recorded using de-identified letter and number combinations. (e.g./ P1, P2 etc). The Likert scales were tabulated to assess the impact of workplace coaching.

## Results

### Training and program delivery

Twelve fitness instructors undertook the initial workshop (10 females, mean age 42 ± 10 years; mean 8.3 ± 4.7 years’ experience in the fitness industry) who were delivering exercise with the community centre. All 12 instructors had a provincial designation as a registered fitness instructor. Ten of these instructors also had a bachelor’s degree; three of these in kinesiology and in addition two of these instructors had master’s level qualification in exercise science or exercise physiology.

The FAME program ran for 1 hour on 2 days a week led by two trained instructors at each session. Four instructors taught regularly and were present for the workplace coaching. Two other instructors were available for relief and taught in the program as needed.

### Audit results

Program fidelity was generally high based on our TIDier audit checklist, but three of the 14 items had some non-adherence. The three items identified as not meeting audit criteria were; Item 4.2: intensity monitoring, item 3.7: discussing educational tips (e.g., healthy eating, stress management) and Item 7: adequate space. Potential solutions were discussed with the fitness instructors to rectify these problems. Program fidelity improved over the course of the audits. Difficulties in using personal carers in class was identified as a challenge at the second audit, although this was not on the original checklist.

### Timing and content of the workplace coaching

The TIDier checklist audit results informed the subsequent workplace coaching session in terms of learning objectives, content and preferred format for delivery. This was done in collaboration between the fitness instructors and the auditor. Multiple educational strategies were used in the workplace coaching, which included development of new written and equipment-based resources and practical components. The written material was subsequently included in the workshop manual for future delivery of training to the next cohorts of fitness instructors.

The first workplace coaching session addressed Item 4.2 and included intensity monitoring information (written information about calculating target heart rates) and use of heart rate monitors. To meet the criteria of providing educational tips (Item 3.7), a more detailed script for the educational tips was used, with coaching on their use in the class.

The second workplace coaching session included demonstrations of how to instruct carers to assist with management of their client in the class (Item 11). This included where to stand for balance activities and how and where to hold their client in the class. With the high turnover of carers attending with the people with stroke, a handout was requested by the fitness instructors to give to the carers about how to support their person in the class. This was developed by the research team and included in the manual in the subsequent versions.

The third workplace coaching session addressed TIDier audit checklist item 3.7 and included a discussion of why the educational tips were still not being used. The fitness instructors commented that it was difficult to include the information and exercises in the same session and suggested the provision of handouts for the class participants.

The fourth workplace coaching session included a visit to negotiate with management to move the program to a larger space to meet the TIDier checklist audit criteria 7.

### Survey themes

Six of the 12 instructors that attended the workshop had opportunity to teach the FAME program over the first year. All these six instructors completed a survey at 1 year to provide feedback on the FAME program implementation. While the other 6 instructors applied their FAME knowledge to other programs they delivered, they were not specifically evaluated. Three themes relating to the program fidelity emerged from the open-ended survey data. These themes are ‘program delivery – highlights and challenges’, ‘active ingredients’, and ‘confidence from coaching’.

### ‘Program delivery - highlights and challenges’

In general, the instructors found that the program was well received and could describe gains made for the participants with stroke, a chronic disease population that has more physical and cognitive needs than typically seen by fitness instructors: “I see such an improvement with the participants. They all have complemented us on the class and how they are enjoying it.” (P1) and “seeing the improvements in the people in their balance and stamina was gratifying.” (P6) Five people commented on the small exercise space when asked about challenges in delivering the program. Another person described the challenge of delivering a program to people of different abilities, especially as some people needed a caregiver to support them to participate. Five of the six respondents indicated that they had made changes to the program, although only two provided details which within the scope of the program (e.g., progressing the exercises as people changed ability, adding arm exercises). Neither of these changes altered program fidelity.

### ‘Active ingredients’

The explicit identification of 7 essential “active ingredients” which was part of the Tidier audit was helpful to maintain fidelity and it appeared that respondents were delivering the program in line with the program principles as intended; they identified several components of the program itself that were working well, including the program plan, and the actual exercise components. The resources provided in the training workshop were “helpful to keep all of the instructors on the same direction.” (P5). One respondent stated that the high level of evidence of the program was a major reason why they had not changed the program delivery. Ongoing commitment to program delivery was noted by one respondent; “I see the importance of this program in the community and would like to be more involved teaching [it] in the future.” (P2).

### ‘Confidence from coaching’

All six respondents agreed or strongly agreed that they were confident to deliver the program and that their confidence had improved over the year since initial training. Five out of six respondents agreed or strongly agreed that they understood common impairments for people with stroke, with one neutral response. All respondents agreed or strongly agree that they understood the exercise benefits for people after stroke. Responses related to program principles of repetition and progression were positive (agreed or strongly agreed), with only one neutral response. Five out of six respondents also agreed or strongly agreed that their confidence in physically supporting people within the class had improved, with one neutral response.

## Discussion

Complex interventions are hard to reproduce [3] and identifying the ‘active ingredients’ is challenging [17]. Essential elements of complex health interventions have been identified [18] and appear in our adapted TiIDier checklist from the multiple clinical trials that established the intervention efficacy. The same two physical therapists identified the active ingredients (Table 1) and came to consensus of the items informed by a series of randomized controlled trials by our research team which led to the development of FAME. For example, Marigold et al. [11] incorporated an agility/balance/functional strengthening component which reduced falls, while the paper by Pang [12] added a fitness component with intensity monitoring which improved cardiovascular fitness. Both prior trials used high repetitions to produce these benefits, and in addition, there is growing evidence [19] that supports repetitions. A focus on normal movement control is also based in literature on motor control recovery after stroke.

The application of the TIDier framework required us to identify and describe the ‘active ingredients’ in sufficient detail to facilitate the implementation of the intervention as intended, as well as to enable evaluation of program fidelity. The future use of this tool in supporting and evaluating fidelity during wider scale replication warrants further exploration.

The high levels of implementation fidelity for the delivery of an exercise program for people after stroke was supported by the use of a checklist at multiple points within this project. Initially, the checklist was used within the training workshop to clarify the key ‘active ingredients’ that were required for program fidelity to the instructors. Second, the checklist was used as a basis for the audit and feedback process within the workplace visits, enabled the quantitative measurement of fidelity and supported demonstrable improvements in fidelity over time. Assessing Implementation fidelity is vital for determining effectiveness, feasibility and variation in the intervention delivery [20].

This is the first time that the TIDier checklist [14] has been used in the context of an implementation fidelity evaluation. The TIDier framework has been used in several ways outside its original purpose of providing clinical trial replication detail, from feasibility to intervention development [21]. The headings of the TIDier checklist matched well with the needs for implementation evaluation, especially with the large emphasis on tailoring and modification which are key to group programs that require individualized progression. As well, the checklist provided a format for the fidelity criteria to be described in actionable detail; this detail was not available in the previously developed program material. As the format of use of this checklist was as a workplace audit (and not as part of journal review) we had added two extra headings. Firstly, some of the criteria were not able to be viewed during the class (e.g., adequate screening procedures) and so a topic related to pre-delivery of the program was included. Another heading that identified criteria for feedback or use n workplace coaching was also added. Both additions enhanced the use of the checklist as an audit tool.

While audit and feedback is effective in changing health professional behaviour in health settings [22], this is the first to our knowledge for its use in a community setting. The current study expands the use of audit and feedback as an implementation tool to be used in community settings for complex interventions. Evidence from our worksite audits and fitness instructor survey data demonstrated improvements in instructor confidence in delivering the exercise program, as well as working with carers of the participants with stroke. In addition, there was high fidelity in delivering the “active ingredients” of the exercise intervention. As such, it is likely that this program may produce benefits similarly to those seen in published research in this area. However, description of changes in functional ability of the people with stroke who attended the exercises program or qualitative feedback from these clients is not presented here and is the focus of a separate paper.

### Strengths and limitations

It is a strength of this study that there were multiple iterations of audit and workplace coaching using the TIDier audit checklist to improve fidelity of the program over a year. Transferability may be limited because data was only collected at one site with a small number of participants.

## Conclusion

Implementing complex community-based exercise requires the people delivering exercise be trained to ensure that all the active ingredients of the program are addressed. Through defining, describing and embedding the ‘active ingredients’ (program principles) in training and then using an audit and feedback process to measure fidelity and inform ongoing learning, we have demonstrated that evidence-based exercise can be delivered with fidelity within a community centre. A novel checklist based on the TIDier framework supported program implementation and fidelity evaluation. Other program deliverers may find this TIDier adapted checklist useful to measure their own program fidelity.

## Supplementary information

**Additional file 1.** Survey for Exercise Instructors.

**Additional files 2: Table S1.** TIDier adapted checklist with examples.

## Data Availability

The datasets analysed during the current study available from the corresponding author on reasonable request.
