# Supplementary material for: Evaluation and facilitation of intervention fidelity in community exercise programs through an adaptation of the TIDier framework
Source: BMC Health Serv Res. 2020 Jan 30;20:68. doi: 10.1186/s12913-020-4919-y (PMC6993417; doi:10.1186/s12913-020-4919-y)
Supplement: Supplementary file 1 — Additional file 1. Survey for Exercise Instructors. [file 12913_2020_4919_MOESM1_ESM.pdf]

Additional file 1

Survey for Exercise Instructors

Q1 Have you been teaching into the FAME program? (Yes/No)

Q2 What is working well in the FAME classes?

Q3 What challenges are you finding with the FAME classes?

Q4 Are you running the program as it was taught or have you made some modifications?
Yes, I am running it as per the manual, No, I have made some changes (Please describe) _

	Strongly Disagree	Disagree	Neutral	Agree	Strongly agree
I am confident that i can run a class for people with stroke	O	O	O	O	O
I have improved in confidence in delivering the FAME program since the training last year	O	O	O	O	O
. I understand how common impairments affect exercise abilities for people with stroke	O	O	O	O	O
I understand the benefits of exercise for people with stroke	O	O	O	O	O
I understand the principle of exercise progression for people with stroke	O	O	O	O	O
I understand the important principle of repetition of exercise for people with stroke	O	O	O	O	O
I understand the important principle of repetition of exercise for people with stroke	O	O	O	O	O
I think the participants like the education tips	O	O	O	O	O

I find the education tips easy to include in the class	O	O	O	O	O
I think the participants like the activity monitors	O	O	O	O	O
I find the activity monitors easy to include in the class	O	O	O	O	O
I am confident in giving instructions to the carers of participants in the class	O	O	O	O	O
I feel comfortable giving physical support to clients in the class who need it	O	O	O	O	O

Q6 I would like more training (Yes/No)

Q7 If yes, please describe the format desired

- ☐ Workplace coaching,
- ☐ Workshop outside of classes,
- ☐ Online learning or Other ____

Q8 Would you like to have the hospital physical therapists visit or provide more information for new clients?

Q9 Would you like to visit the hospital physiotherapists regularly (e.g. once or twice a year)?

Q10 Would you like to attend the next round of FAME instructor workshops for fitness instructors with a view to becoming a FAME instructor educator (in partnership with a physiotherapist)?

Q11 Please provide any additional comments below:

____
