# Supplementary material for: Evaluation and facilitation of intervention fidelity in community exercise programs through an adaptation of the TIDier framework
Source: BMC Health Serv Res. 2020 Jan 30;20:68. doi: 10.1186/s12913-020-4919-y (PMC6993417; doi:10.1186/s12913-020-4919-y)
Supplement: Supplementary file 2 — Additional files 2: Table S1. TIDier adapted checklist with examples. [file 12913_2020_4919_MOESM2_ESM.docx]

Table S1. TIDier adapted checklist with examples

| **Item number** | **Item description** | **Yes/No** |
| --- | --- | --- |
|  | **BRIEF NAME** | **Y** |
| **1.** | Program marketed as the ‘FAME exercise program’ | Y |
|  | **WHY** |  |
| **2.** | Justifies activities to participants based on the first 4 principles (need for progression, repetition, intensity and normal movement patterns). | Y |
|  | **WHAT** |  |
| **3.** | 3.1 Progressions – participants work at increasingly more challenging levels  3.2 Repetition – during each 5-minute station, high levels of repetition are seen  3.3 Intensity – a moderate level of intensity  3.4 Normal movement patterns -e.g., equal weight bearing, good posture  3.5 Core Components – each class has balance, strength and agility/fitness stations  3.6 Encouragement  Verbal encouragement at least 3 times  Acknowledges success/progression for each participant  3.7 Education tip included | Y  Y  N  Y  Y  Y  Y  N |
| **4.** | Procedures:  4.1 All Core components included  Balance activities challenge balance safely  Fitness activities at moderate intensity  Functional strengthening performed safely  Functional strengthening at moderate intensity  Resistance  Repetitions  4.2 Intensity monitored at 3 timepoints  Heart rate  Perceived exertion  4.3 More than half the class in standing  If clients must sit, they are given a sitting exercise to go on with  Intensity modified to reduce any abnormal movement | Y  Y  Y  Y  Y  Y  Y  N  N  Y  Y  Y |
|  | **WHO PROVIDED** |  |
| **5.** | Intervention provided by trained instructor | Y |
|  | Ratio of instructor to participants no more than 1:5 | Y |
|  |  |  |
|  | **HOW** |  |
| **6.** | Uses verbal cues for safety  Uses verbal cues for changing intensity  Verbal cues to encourage good form  Uses tactile cues for safety (including spotting by instructor)  Uses tactile cues for changing intensity  Sets up environment for safety (including position of instructor)  Sets up environment for intensity  Sets up environment and choosing exercises for success  Demonstrates exercises to facilitate good exercise form | Y  Y  Y  Y  Y  Y  Y  Y  Y |
|  | **WHERE** |  |
| **7.** | Space adequate for stations  Space allows safe client flow | Y/N  Y |
|  | **WHEN and HOW MUCH** |  |
| **8.** | Classes presented on two non-consecutive days | Y |
|  | Classes last 60 minutes | Y |
|  | Warm up, Core components and cool down stretch  Balance activities 15 minutes  Functional strength 15 minutes  Fitness activities 15 minutes | Y  Y  Y  Y |
|  | **TAILORING** |  |
| **9.** |  |  |
| **10.** |  |  |
| **11** | Personal carers appropriately utilized in class | N |
| **12.** | **ACTUAL**  The extent to which the intervention was delivered as planned.  If no, please describe | **Y** |
| **13** | **ASPECTS RELATED TO PRE-DELIVERY**  Selection of appropriate clients | **Y** |
| **14** | **DEBRIEF TOPICS**  Instructors review list of topics provided by auditor |  |

Comments:

3.6. Instructors were very positive and enthusiastic in their instruction, and ensured that they facilitated the group as a whole, as well as providing individual directions and spotting to participants

3.7. No educational tip was provided

4.2. Intensity via heart rate or Borg exertion scale was not regularly used to check the exertion or how hard participants were working, although instructors did check with participants asking “how are you doing”.

5.0. Two lower functioning persons with stroke each came with a young personal carer who assisted a small amount but it was clear that they did not know what role they could play in the class and for the most part, sat on the sidelines. These carers played a role in helping with transfers and mobility in the home and thus, expanding the carer’s role in the class could be explored.

7.0 The space for this class was relatively small and ideally could have been larger. However, the instructors made very good use of the small space, ensuring that participants were appropriately spaced and had a small portable ballet bar set up in the middle to help with balance exercises.
